# The Genetics of Atrial Fibrillation: From Molecular Mechanisms to Precision Medicine

**DOI:** 10.7759/cureus.109864

**Published:** 2026-05-29

**Authors:** Dilip D Hinge, Abhijeet Shelke, Ramesh Kawade, Satish Patil

**Affiliations:** 1 Molecular Biology and Genetics, Krishna Vishwa Vidyapeeth (Deemed to be University), Karad, IND; 2 Cardiology, Krishna Vishwa Vidyapeeth (Deemed to be University), Karad, IND; 3 Microbiology, Krishna Vishwa Vidyapeeth (Deemed to be University), Karad, IND

**Keywords:** atrial fibrillation, cardiac arrhythmia, genetics, ion channels, precision medicine, transcription factors

## Abstract

Atrial fibrillation (AF) is the most common sustained cardiac arrhythmia, characterized by rapid and disorganized electrical activity in the atria. Although traditionally attributed to aging and structural remodeling secondary to cardiovascular comorbidities, AF has a well-recognized heritable component, with genetic factors contributing substantially to disease susceptibility. This review systematically evaluates the molecular landscape of AF, focusing on genetic mutations and their functional consequences in disease development. AF-associated genes are organized into four functional modules: (1) ion channelopathies (*SCN5A*, *KCNQ1*, *KCNH2*, *KCNA5*); (2) structural myopathies (*TTN*, *MYL4*, *GJA5*, *COL1A1*); (3) transcriptional regulators (*PITX2, GATA4, TBX5, NKX2-5, GATA6,* *ZFHX3*); and (4) calcium handling proteins (RYR2, CASQ2, JPH2, ANKB, PLN, TRDN). For each gene, population prevalence and penetrance of associated variants are discussed alongside the specific molecular and biophysical consequences of causative mutations. By elucidating how these protein alterations converge on shared electrophysiological and structural substrates, we provide a framework for transitioning from generalized therapeutic strategies to precision medicine approaches in cardiac electrophysiology.

## Introduction and background

Atrial fibrillation (AF) remains the most prevalent sustained cardiac arrhythmia worldwide, contributing substantially to morbidity and mortality through increased risks of stroke, heart failure, cardiovascular death, coronary artery disease, and dementia [1-4]. The prevalence of AF increases exponentially with age, affecting approximately 1-2% of the general population and up to 10% of individuals aged over 80 years [5]. The traditional paradigm attributed AF primarily to age-related structural remodeling and comorbidity-driven atrial changes, conceptualized as a cycle in which sustained AF promotes further atrial remodeling that perpetuates the arrhythmia [6].

Landmark epidemiological studies have demonstrated significant familial aggregation of AF, with first-degree relatives of AF patients exhibiting approximately a 40% increased risk of developing the arrhythmia [7]. This observation has catalyzed a paradigm shift towards understanding the genetic architecture underlying AF susceptibility. Heritability estimates suggest that genetic factors account for up to 62% of AF risk, particularly in early-onset cases occurring before age 60; this figure derives from twin-based biometric modelling and represents broad-sense heritability, encompassing all genetic variance including rare variants, common variants, and gene-gene interactions, with the caveat that shared environmental factors in twin studies are difficult to fully decouple [8].

The genetic landscape of AF encompasses both rare variants with high penetrance, typically identified in familial AF cohorts, and common variants with modest effect sizes (odds ratios (ORs) typically 1.3-1.8) discovered through genome-wide association studies (GWAS). A central challenge, the heritability gap, is that identified common GWAS variants collectively explain only a small fraction of the total familial clustering of AF compared with rare, high-penetrance mutations. This tension underpins the translational discussion throughout this review [9]. Over the past decade, genomic exploration has evolved from studying rare, high-penetrance mutations in monogenic AF to identifying over 160 common risk loci through large-scale GWAS [10]. These studies reveal that genetic risk for AF is distributed across genes regulating cardiac action potential, sarcomeric structure, and atrial developmental identity [11]. The 4q25 locus, harboring PITX2, consistently emerges as the strongest genetic signal, linking altered transcriptional programming of the left atrium to arrhythmia susceptibility [12,13].

This review synthesizes current knowledge across four functional modules, ion channelopathies, structural myopathies, transcriptional regulators, and calcium-handling proteins, examining how specific amino acid substitutions and protein truncations alter the biophysics of atrial conduction and provide molecular substrates for electrical and structural remodeling. Critically, each gene discussion is contextualized by its estimated population prevalence, penetrance, and the clinical scenarios in which genetic knowledge currently changes patient management. The clinical implications, including appropriate indications for targeted genetic testing, genotype-guided therapy selection, and future precision medicine directions, are discussed.

## Review

Ion channel mutations and electrophysiological remodeling

Ion channels represent the fundamental molecular machinery governing cardiac electrophysiology. These transmembrane proteins regulate the flux of sodium, potassium, and calcium ions that orchestrate the cardiac action potential. Genetic variants affecting ion channel function constitute a major category of AF-associated mutations, directly impacting action potential duration, conduction velocity, and refractory periods. The developmental expression of these channels is governed by transcription factors such as PITX2, establishing atrial electrical identity during embryogenesis [14]. Disruption of this developmental programming creates an inherent substrate for arrhythmogenesis even before clinical manifestation.

Sodium Channel Dysfunction: SCN5A Mutations

The *SCN5A *gene encodes the alpha subunit of the cardiac voltage-gated sodium channel (Nav1.5), responsible for the rapid upstroke of the cardiac action potential (phase 0). *SCN5A* mutations demonstrate remarkable phenotypic heterogeneity, associated with multiple cardiac disorders including long QT syndrome, Brugada syndrome, cardiac conduction disease, and AF [15]. Pathogenic *SCN5A* variants are identified in approximately 2-5% of patients with early-onset lone AF, with higher yields in familial cohorts [16]. The functional consequences can be broadly categorized into trafficking defects, gating abnormalities, and gain-of-function mutations.

Trafficking defects: Trafficking mutations, exemplified by the R1432G variant, result in retention of mutant channel proteins within the endoplasmic reticulum, preventing translocation to the plasma membrane [16]. This produces a functional deficiency of sodium channels at the sarcolemma, severely compromising depolarization capacity and normal impulse propagation [16,10]. The G1743R variant represents another trafficking-defective mutation, frequently associated with overlapping syndromes involving both Brugada syndrome and progressive cardiac conduction disease [17]. The resultant reduction in available sodium current (INa) decreases conduction velocity and creates a substrate for reentrant arrhythmias.

Gating defects: Gating mutations affect the voltage-dependent activation and inactivation kinetics of sodium channels without necessarily impacting protein trafficking. The T187I variant exemplifies this class, producing a negative shift in steady-state inactivation that reduces channel availability during the action potential [18,19]. This mutation is associated with familial sick sinus syndrome and atrial arrhythmias [18]. The altered channel kinetics disrupt precise temporal coordination required for synchronized atrial depolarization, creating conditions favorable for electrical fragmentation and AF initiation.

Gain-of-function mutations: Certain *SCN5A* mutations produce gain-of-function effects through incomplete channel inactivation, generating a persistent inward sodium current (late INa). The K1477N and E1784K variants exemplify this mechanism, creating a persistent sodium current during phases 2 and 3 of the action potential [16,17]. This abnormal sodium influx prolongs action potential duration, generates early afterdepolarizations (EADs), and provides triggers for premature atrial activity that can initiate AF episodes. The dual consequences of altered repolarization and triggered activity make these mutations particularly arrhythmogenic.

Potassium Channel Dysfunction: Alterations in Repolarization

Potassium channels mediate cardiac repolarization and determine the effective refractory period of atrial tissue. Multiple potassium channel genes have been implicated in AF pathogenesis, with mutations producing either gain-of-function or loss-of-function effects that alter repolarization kinetics.

*KCNQ1* gain-of-function mutations: The *KCNQ1* gene encodes the alpha subunit of the slow delayed rectifier potassium channel (IKs), critical for cardiac repolarization. Gain-of-function mutations in *KCNQ1* represent the most common potassium channel defect associated with AF. The *S140G* mutation serves as a prototypical example, causing premature channel activation and enhanced potassium conductance. This increased outward current accelerates repolarization, markedly shortening the atrial effective refractory period (AERP) [20]. The abbreviated AERP allows atrial tissue to recover excitability more rapidly, facilitating the maintenance of high-frequency reentrant circuits characteristic of AF. *KCNQ1* gain-of-function variants are identified in approximately 3-5% of familial AF kindreds [21].

Loss-of-function mutations and repolarization prolongation: Loss-of-function mutations in repolarizing potassium channels prolong action potential duration and also predispose to AF through different mechanisms. Mutations in KCNH2 (encoding the rapid delayed rectifier IKr), such as T895M, reduce potassium current and prolong repolarization [16]. This creates spatial heterogeneity in repolarization across the atria, increasing dispersion of refractoriness and promoting reentry. Additionally, prolonged action potentials enhance calcium influx, potentially triggering calcium-dependent afterdepolarizations.

*KCNA5* and the ultra-rapid delayed rectifier: The *KCNA5* gene encodes Kv1.5 channels responsible for the atrial-specific ultra-rapid delayed rectifier current (IKur). This current significantly contributes to atrial repolarization but has minimal expression in ventricles, making *KCNA5* an atrial-selective target [22]. Loss-of-function mutations, such as E375X and T527M, reduce IKur density, paradoxically predisposing to AF despite prolonging atrial action potential duration [23]. The mechanism likely involves enhanced heterogeneity of repolarization and increased susceptibility to triggered activity. The atrial-specific expression of *KCNA5* makes it an attractive potential therapeutic target for AF management without ventricular effects.

Structural protein mutations and atrial remodeling

Beyond ion channel dysfunction, structural protein abnormalities constitute a major pathway to AF development. Mutations affecting sarcomeric proteins and extracellular matrix components lead to atrial cardiomyopathy, characterized by atrial dilatation, fibrosis, and conduction abnormalities. These structural changes create the anatomical substrate necessary for sustained reentrant arrhythmias.

Sarcomeric Protein Mutations

Titin (TTN) truncating variants: Titin, encoded by TTN, represents the largest protein in the human body, extending from the Z-disc to the M-line of the sarcomere and providing structural stability and passive tension [24]. Truncating variants in TTN (TTNtv), which produce premature termination codons, are found in approximately 1% of the general population but show significant enrichment, up to 18%, in patients with atrial cardiomyopathy and AF [25-28]. These mutations compromise sarcomeric integrity, leading to atrial dilatation and increased wall stress [26]. The resulting mechanical dysfunction triggers compensatory fibrosis, creating slow conduction zones and anchoring reentrant circuits. Importantly, TTN-associated AF often presents as isolated atrial disease without significant ventricular involvement, suggesting differential isoform expression or functional redundancy between chambers [27].

Myosin light chain 4 (MYL4): MYL4 encodes the atrial-specific myosin light chain, a regulatory component of the myosin complex uniquely expressed in atrial tissue [29]. The E11K mutation in MYL4 alters the protein's isoelectric point and calcium sensitivity, disrupting normal atrial contractility [30]. This functional impairment leads to atrial dilatation and electromechanical dysfunction. The atrial-specific expression pattern of MYL4 explains why mutations predominantly manifest as isolated AF without ventricular involvement. MYL4 variants demonstrate high penetrance in familial AF pedigrees, with affected individuals typically developing AF in the fourth or fifth decade of life [31].

Extracellular Matrix Remodeling

Gap junction proteins, GJA5: Connexin 40 (Cx40), encoded by GJA5, form gap junctions that mediate direct electrical coupling between atrial myocytes [32]. The G38D mutation in GJA5 impairs gap junction assembly and electrical conductance, creating zones of reduced intercellular coupling [33]. This heterogeneous connectivity slows atrial conduction velocity and increases anisotropy, both critical determinants of reentrant circuit formation [34]. Reduced Cx40 expression correlates with the degree of atrial fibrosis in AF patients, suggesting a mechanistic link between connexin dysfunction and structural remodeling [35].

Collagen Type I, COL1A1: The *COL1A1* gene encodes type I collagen, the predominant structural protein of the cardiac extracellular matrix [36]. The -1997G/T polymorphism in the COL1A1 promoter region, affecting an Sp1 binding site, increases collagen gene transcription and protein production [37]. This genetic variant is associated with increased atrial fibrosis, characterized by excessive interstitial collagen deposition separating myocyte bundles [38]. The resultant structural heterogeneity creates preferential conduction pathways and anatomical obstacles that anchor reentrant wavelets [39]. Fibrotic tissue also exhibits reduced electrical coupling and serves as an electrical barrier, contributing to conduction slowing and fractionated electrograms observed during electrophysiological mapping of AF patients [40].

Transcriptional regulation and developmental programming

Transcription factors represent master regulators that coordinate the expression of multiple genes simultaneously, establishing and maintaining cardiac chamber identity and electrical properties. Mutations in cardiac transcription factors do not disrupt single proteins but fundamentally reprogram atrial gene expression networks, predisposing to AF through pleiotropic mechanisms.

PITX2: The Master Regulator of Left Atrial Identity

The *PITX2* gene on chromosome 4q25 represents the most robust genetic locus for AF identified through GWAS, with single nucleotide polymorphisms (SNPs) such as rs2200733 and rs10033464 conferring substantial AF risk (OR ~1.7-2.0 per risk allele) [12,13]. *PITX2* encodes a paired-like homeodomain transcription factor critical for left-right asymmetry during cardiac development [41]. These risk SNPs, located in non-coding regions, reduce PITX2 expression levels in adult atrial tissue rather than altering protein sequence [20].

Reduced PITX2 expression leads to a molecular remodeling process in which the left atrium loses its characteristic gene expression profile [42]. Specifically, PITX2 deficiency downregulates KCNQ1 and other repolarizing potassium channels while upregulating calcium-handling proteins and pacemaker channels [21]. This transcriptional remodeling shortens the atrial effective refractory period and increases automaticity, creating an electrophysiological substrate conducive to AF. Additionally, PITX2 normally suppresses the development of myocardial sleeves extending into pulmonary veins; its reduction is associated with abnormal muscularization of these vessels, creating ectopic foci that trigger AF [43]. Animal models demonstrate that Pitx2 haploinsufficiency produces spontaneous AF and recapitulates key features of human disease [44].

GATA4: Structural Integrity and Chamber Morphogenesis

*GATA4* encodes a zinc-finger transcription factor essential for cardiac development and maintenance of chamber-specific gene expression [45]. The p.Gly296Ser (G296S) mutation disrupts the DNA-binding domain of *GATA4*, impairing its ability to activate target genes involved in atrial wall formation and intercellular junction assembly [46]. Functional studies demonstrate that this mutation results in reduced expression of genes encoding gap junction proteins and extracellular matrix components, leading to increased atrial fibrosis and impaired electrical coupling [47]. The structural consequences manifest as progressive atrial dilatation and conduction abnormalities, with affected individuals typically developing AF in their thirties or forties. *GATA4* mutations also associate with atrial septal defects, suggesting broader roles in atrial structural organization [48].

TBX5: Conduction System Development

*TBX5* encodes a T-box transcription factor crucial for cardiac chamber specification and conduction system formation [49]. The p.Gly125Arg (G125R) mutation in *TBX5* produces a gain-of-function effect, enhancing DNA binding affinity and dysregulating the expression of target genes involved in electrical conduction [50]. This mutation disrupts normal sinoatrial node development and creates heterogeneity in atrial conduction properties, manifesting as early-onset familial AF often accompanied by bradycardia [51]. TBX5 haploinsufficiency causes Holt-Oram syndrome, characterized by upper limb abnormalities and congenital heart defects, including atrial septal defects and conduction disease [52]. The association between *TBX5* variants and AF underscores the developmental origins of adult arrhythmia susceptibility.

NKX2-5: Chamber Septation and Cellular Homeostasis

*NKX2-5* represents a critical homeodomain transcription factor regulating cardiomyocyte differentiation and chamber septation [53]. The p.Asn197Lys (N197K) mutation disrupts the homeodomain responsible for DNA binding, impairing NKX2-5-dependent gene activation [54]. This mutation is associated with atrial septal defects, progressive atrioventricular conduction block, and early-onset AF [55]. The structural consequences include atrial wall thinning and compromised electrical insulation between cardiac chambers, creating substrates for both reentrant circuits and abnormal electrical communication between atria and ventricles [56]. *NKX2-5* mutations demonstrate variable expressivity, with some family members developing isolated conduction disease while others present with complex structural heart disease.

GATA6: Pulmonary Vein Myocardial Sleeve Formation

*GATA6*, another member of the GATA transcription factor family, specifically regulates the developmental identity of pulmonary vein myocardial sleeves [57]. The p.Ala178Gly (A178G) mutation impairs *GATA6* transcriptional activity, leading to abnormal extension of atrial myocardium into pulmonary veins [58]. This architectural anomaly creates hyperexcitable tissue with enhanced automaticity and triggered activity [59]. Catheter ablation studies have established pulmonary vein isolation as an effective AF therapy, directly validating the importance of pulmonary vein triggers in AF pathogenesis [60]. *GATA6* mutations thus provide a genetic explanation for the spontaneous firing from pulmonary veins that initiates paroxysmal AF in many patients.

ZFHX3: Stress Response and Atrial Remodeling

*ZFHX3* (also known as ATBF1) encodes a large zinc-finger homeodomain transcription factor involved in stress response and inflammatory signaling [61]. The rs2106261 SNP near *ZFHX3* is associated with increased AF risk in multiple populations [62]. Functional studies suggest that altered ZFHX3 expression impairs the atrial stress response, leading to enhanced susceptibility to oxidative damage and inflammatory remodeling [63]. Reduced ZFHX3 function correlates with atrial enlargement and increased fibrosis [64]. The *ZFHX3* locus demonstrates particularly strong associations with cardioembolic stroke, suggesting that ZFHX3-related AF may exhibit higher thrombogenicity or greater hemodynamic compromise [65]. It should be noted that ZFHX3 is primarily a GWAS-identified stress-response factor, and its mechanistic evidence base is less complete than that of the developmental transcription factors discussed above.

Calcium-handling abnormalities and triggered activity

Intracellular calcium handling represents a critical determinant of cardiac excitation-contraction coupling and cellular electrophysiology. Abnormalities in calcium cycling proteins can generate spontaneous electrical activity through delayed afterdepolarizations (DADs) and early afterdepolarizations (EADs), serving as triggers for AF initiation.

Ryanodine Receptor 2 (RYR2)

*RYR2* encodes the cardiac ryanodine receptor, the primary calcium release channel of the sarcoplasmic reticulum [66]. The p.Arg4497Cys (R4497C) mutation and other *RYR2* variants increase the channel's sensitivity to cytosolic calcium, producing spontaneous calcium release events during diastole [67]. These abnormal calcium transients activate the sodium-calcium exchanger (NCX) in its forward mode, generating a depolarizing inward current that manifests as DADs [68]. When DADs reach the threshold, they trigger premature atrial complexes that can initiate AF. *RYR2* mutations are classically associated with catecholaminergic polymorphic ventricular tachycardia (CPVT), but atrial arrhythmias, including AF, represent common manifestations, particularly during adrenergic stimulation [69].

Calsequestrin 2 (CASQ2)

*CASQ2* encodes calsequestrin 2, the primary calcium-buffering protein within the sarcoplasmic reticulum lumen [70]. The p.Asp307His (D307H) mutation impairs the calcium-binding capacity of calsequestrin, leading to sarcoplasmic reticulum calcium overload [71]. This overload increases the open probability of RYR2 channels, producing spontaneous calcium waves similar to those caused by direct *RYR2 *mutations [72]. *CASQ2* mutations cause autosomal recessive CPVT but also associate with atrial arrhythmias through calcium-dependent triggered activity affecting both atrial and ventricular myocardium [73].

Junctophilin 2 (JPH2)

*JPH2* encodes junctophilin 2, a structural protein that anchors the sarcoplasmic reticulum to the plasma membrane, maintaining proper spatial organization of calcium release units [74]. The p.Glu169Lys (E169K) mutation disrupts this structural tethering, causing misalignment between L-type calcium channels and RYR2 receptors [75]. This geometric disorganization desynchronizes excitation-contraction coupling and promotes spontaneous calcium release events [76]. *JPH2* mutations associate with hypertrophic cardiomyopathy and AF, suggesting that structural integrity of calcium handling machinery significantly impacts arrhythmia susceptibility [77].

Ankyrin-B (ANKB)

*ANKB* encodes ankyrin-B, an adaptor protein that localizes and stabilizes ion channels and transporters at specific membrane domains [78]. The p.Ser646Phe (S646F) mutation impairs targeting of the sodium-calcium exchanger and sodium-potassium ATPase to their functional membrane locations [79]. Loss of normal NCX localization reduces calcium extrusion capacity, leading to intracellular calcium overload and triggered activity [80]. Ankyrin-B syndrome manifests as a complex phenotype including sinus node dysfunction, AF, and ventricular arrhythmias, reflecting the protein's diverse roles in cardiac electrophysiology [81].

Phospholamban (PLN)

*PLN* encodes phospholamban, a key regulator of the sarcoplasmic reticulum calcium ATPase (SERCA2a) responsible for calcium reuptake [82]. The p.Arg14del (R14del) mutation, particularly prevalent in Dutch populations (carrier frequency ~0.3%), causes constitutive inhibition of SERCA2a, slowing calcium reuptake and prolonging cytosolic calcium transients [83]. This impaired calcium removal predisposes to both diastolic calcium accumulation, triggering DADs, and incomplete recovery before the next action potential generating EADs [84]. PLN mutations are primarily associated with dilated cardiomyopathy and heart failure, but AF represents a common comorbidity, likely secondary to both calcium handling abnormalities and structural remodeling [85].

Triadin (TRDN)

*TRDN* encodes triadin, a component of the junctional sarcoplasmic reticulum complex that modulates RYR2 activity [86]. The p.Thr59Arg (T59R) mutation destabilizes the RYR2-triadin-calsequestrin macromolecular complex, increasing the propensity for spontaneous calcium release [87]. This structural destabilization creates stochastic calcium release events that generate DADs and trigger premature atrial activity [88]. *TRDN* mutations associate with both CPVT and AF, highlighting the shared mechanisms of calcium-dependent triggered activity in different cardiac chambers [89].

Table 1 summarizes all discussed genes, their key variants, molecular mechanisms, and associated clinical phenotypes across the four functional modules.

**Table 1:** Summary of AF-associated genetic mutations by functional module AERP: atrial effective refractory period; ASD: atrial septal defect; CPVT: catecholaminergic polymorphic ventricular tachycardia; DAD: delayed afterdepolarization; DCM: dilated cardiomyopathy; EAD: early afterdepolarization; ECC: excitation-contraction coupling; HCM: hypertrophic cardiomyopathy; LQTS: long QT syndrome; NCX: sodium-calcium exchanger; PV: pulmonary vein; SR: sarcoplasmic reticulum; AF: atrial fibrillation

Module	Gene	Key Variants	Molecular Mechanism	Clinical Phenotype
Ion Channelopathies	SCN5A	R1432G, G1743R, T187I, K1477N, E1784K	Trafficking defects, gating abnormalities, gain-of-function late INa	LQTS, Brugada, AF
Ion Channelopathies	KCNQ1	S140G	Gain-of-function; shortened AERP; facilitates reentry	Familial AF
Ion Channelopathies	KCNH2	T895M	Loss-of-function; prolonged repolarization; dispersion of refractoriness	AF, LQTS
Ion Channelopathies	KCNA5	E375X, T527M	Loss-of-function IKur; atrial-specific repolarization disruption	Familial AF
Structural Myopathies	TTN	Truncating variants	Sarcomeric instability; atrial dilatation; fibrosis	Atrial cardiomyopathy, AF
Structural Myopathies	MYL4	E11K	Disrupted atrial contractility; electromechanical dysfunction	Isolated AF
Structural Myopathies	GJA5	G38D	Impaired gap junction; conduction slowing; anisotropy	AF, atrial fibrosis
Structural Myopathies	COL1A1	-1997G/T promoter	Increased collagen; atrial fibrosis; reentrant substrate	AF
Transcriptional Regulators	PITX2	rs2200733, rs10033464 (non-coding)	Reduced expression; loss of left atrial identity; shortened AERP; PV sleeves	AF (strongest GWAS signal)
Transcriptional Regulators	GATA4	G296S (p.Gly296Ser)	Impaired DNA binding; atrial fibrosis; gap junction loss	AF, ASD
Transcriptional Regulators	TBX5	G125R (p.Gly125Arg)	Gain-of-function; SAN dysfunction; conduction heterogeneity	Familial AF, Holt-Oram
Transcriptional Regulators	NKX2-5	N197K (p.Asn197Lys)	Impaired DNA binding; ASD; AV block; AF	AF, congenital HD
Transcriptional Regulators	GATA6	A178G (p.Ala178Gly)	Abnormal PV myocardial sleeves; ectopic foci	Paroxysmal AF
Transcriptional Regulators	ZFHX3	rs2106261 (GWAS SNP)	Impaired stress response; atrial enlargement; fibrosis	AF, cardioembolic stroke
Calcium Handling	RYR2	R4497C	Spontaneous SR Ca2+ release; DADs; triggered activity	AF, CPVT
Calcium Handling	CASQ2	D307H	Reduced Ca2+ buffering; SR overload; enhanced RYR2 opening	AF, CPVT (AR)
Calcium Handling	JPH2	E169K (p.Glu169Lys)	SR-plasma membrane uncoupling; desynchronized ECC	AF, HCM
Calcium Handling	ANKB	S646F (p.Ser646Phe)	NCX/NaKATPase mislocalization; Ca2+ overload; triggered activity	Ankyrin-B syndrome, AF
Calcium Handling	PLN	R14del (p.Arg14del)	SERCA2a inhibition; Ca2+ accumulation; DADs and EADs	DCM, AF
Calcium Handling	TRDN	T59R (p.Thr59Arg)	Destabilized RYR2 complex; stochastic Ca2+ release; DADs	AF, CPVT

Clinical implications and future directions

Genotype-Phenotype Correlations and Clinical Prevalence

The genetic architecture of AF reveals considerable heterogeneity in both penetrance and clinical presentation. Ion channel mutations typically manifest as early-onset AF, often occurring in the absence of structural heart disease [80]. In contrast, mutations affecting structural proteins or transcription factors frequently associate with progressive atrial cardiomyopathy and may present with mixed phenotypes including heart failure and thromboembolic complications [81]. These differences in presentation define distinct clinical phenotypes that direct genetic evaluation strategies.

Population prevalence and penetrance vary substantially across genes. TTNtv is the most prevalent individually identifiable genetic cause of AF, found in approximately 1% of the general population and 18% of atrial cardiomyopathy patients [28]. *PITX2* common risk variants are highly prevalent, but each confers modest individual risk. In contrast, rare high-penetrance variants in *SCN5A*, *KCNQ1*, *MYL4*, and *NKX2-5* are uncommon but nearly fully penetrant in familial AF pedigrees. Understanding these prevalence and penetrance differences is essential for correctly communicating risk to patients and families.

Genotype also predicts treatment response. Individuals carrying PITX2 risk alleles demonstrate reduced success rates following catheter ablation, possibly due to more extensive atrial remodeling or anatomical complexity of pulmonary vein-atrial junctions [82]. Patients with specific *SCN5A* mutations identified through genotyping may benefit from sodium channel-blocking antiarrhythmic drugs, consistent with guideline recommendations for precision drug selection [83,84]. However, careful clinical and electrophysiological characterization is essential before drug initiation to avoid proarrhythmic effects in patients with coexistent loss-of-function phenotypes such as Brugada syndrome.

Genetic Testing and Clinical Utility

Current guidelines do not recommend routine genetic testing for all AF patients, given the polygenic nature of most cases and limited therapeutic implications [85,90-92]. Whole-genome or whole-exome sequencing is not standard clinical practice for AF. However, targeted multi-gene panel testing is commercially available and clinically indicated in specific scenarios, ordered routinely in inherited arrhythmia clinics and cardiac genetics services at tertiary centres [45]. Panels from providers including Blueprint Genetics Oy (Espoo, Finland), GeneDx, LLC (Stamford, Connecticut, United States), and Invitae Corporation (San Francisco, California, United States) incorporate *SCN5A*, *KCNQ1*, *KCNH2*, *TTN*, *MYL4*, *PITX2*, *GATA4*, *TBX5*, *NKX2-5*, and calcium-handling genes.

Genetic testing should be considered in: early-onset AF (age <40 years) without structural heart disease; familial AF with two or more affected first-degree relatives; AF associated with known cardiac channelopathies or cardiomyopathies; and unexplained sudden cardiac death in a family member where a genetic cause is suspected [86]. The 2020 European Society of Cardiology (ESC) Guidelines and the 2023 American College of Cardiology (ACC)/American Heart Association (AHA)/American College of Clinical Pharmacy (ACCP)/Heart Rhythm Society (HRS) AF Guideline acknowledge the role of genetic evaluation in selected AF patients [91,92]. Identification of pathogenic variants enables cascade screening of relatives, potentially identifying at-risk individuals before clinical manifestation.

Interpretation of genetic variants requires careful consideration of variant frequency in control populations, functional data, segregation with disease in families, and in silico prediction algorithms [87]. Variants of uncertain significance remain common, necessitating longitudinal follow-up and functional validation to clarify pathogenicity.

Precision Medicine Approaches

The molecular insights gained from genetic studies are beginning to translate into precision medicine strategies. Pharmacogenomics may guide antiarrhythmic drug selection based on ion channel genotype. Gene-specific therapies, including antisense oligonucleotides and small-molecule correctors for trafficking-defective channels, show promise in preclinical models as emerging research directions [88]. Polygenic risk scores integrating multiple common variants demonstrate predictive value for AF development and could identify individuals who would benefit from enhanced surveillance or preventive interventions [89]. However, current polygenic risk scores explain only a modest fraction of total familial AF clustering, underscoring the persistent heritability gap and the need for comprehensive variant discovery across diverse populations.

Stem cell-derived cardiomyocytes from patients carrying specific mutations (induced pluripotent stem cells (iPSCs)) provide valuable platforms for proof-of-concept mechanistic studies in familial AF [90]. It is important to note that current iPSC-derived cardiomyocyte models have documented limitations for AF research: they predominantly adopt a ventricular-like electrophysiological phenotype, display immature fetal-like action potential morphology, and lack the atrial-specific properties necessary for rigorous AF modelling. Their current application is best characterized as a mechanistic investigation rather than high-throughput drug screening. Genome editing technologies such as clustered regularly interspaced short palindromic repeats (CRISPR)-Cas9 offer potential for permanent correction of monogenic AF mutations; however, significant challenges specific to the atria, including the need for atrial-targeted viral vectors, efficient in vivo editing, and off-target effects, as well as ethical considerations, mean clinical application remains a future aspiration rather than near-term reality [93].

Systems Biology and Network Medicine

Future progress in AF genetics will require integrating multiple levels of biological organization. Single-variant analyses must be complemented by pathway-based approaches, recognizing that AF results from perturbations in interconnected molecular networks [94]. Integration of genomic data with transcriptomics, proteomics, and metabolomics will provide comprehensive molecular portraits of AF pathophysiology. Machine learning algorithms integrating polygenic risk scores with real-world clinical data, including ECG features and left atrial volume measurements, represent a promising research direction for predicting ablation success and enabling personalized risk stratification, with initial proof-of-concept studies demonstrating feasibility [95]. These future tools are distinguished clearly from currently established clinical practice.

Anticoagulation and Stroke Prevention: Precision Considerations

Stroke prevention through anticoagulation remains a cornerstone of AF management regardless of genetic substrate. Direct oral anticoagulants (DOACs), dabigatran, apixaban, rivaroxaban, and edoxaban, have largely supplanted vitamin K antagonists given their superior safety and efficacy profiles in large randomized trials [72-75]. The emerging class of factor XI inhibitors represents a potentially safer anticoagulant option for patients at high bleeding risk, with preliminary evidence suggesting a more favorable efficacy-to-bleeding ratio compared with existing DOACs; this is particularly relevant for precision therapy in AF [96]. A careful individualized assessment of stroke risk versus bleeding risk is essential, especially in frail elderly patients with AF in whom both thromboembolic and hemorrhagic risks are heightened, and formal frailty evaluation should be incorporated into therapeutic decision-making [97]. Precision medicine approaches to anticoagulation, guided by genetic predictors of bleeding risk, pharmacokinetic variability, and thromboembolic phenotype, represent an important frontier for future research.

Limitations and challenges

Despite remarkable progress, significant gaps remain in our understanding of AF genetics. The heritability gap persists, with identified variants explaining only a fraction of the familial clustering observed epidemiologically. Gene-environment interactions substantially modulate AF risk [98,99], and integrating environmental factors into predictive models remains challenging. The phenotypic complexity of AF, including different temporal patterns (paroxysmal, persistent, permanent) and coexistence with other cardiac conditions, complicates genetic analyses [100].

Most genetic studies have focused on populations of European ancestry, limiting generalizability to other ethnic groups. Expanding genetic studies to diverse populations is essential for equitable precision medicine [101]. Additionally, LMNA (encoding Lamin A/C, a nuclear lamina protein) represents an established AF-associated gene, causing early-onset AF with progressive atrioventricular block, dilated cardiomyopathy, and sudden cardiac death, that does not fit neatly into the four functional modules presented here [51]. Such genes highlight that the molecular classification of AF continues to evolve. This review is a narrative synthesis and did not follow a formal systematic search strategy; future work should apply Preferred Reporting Items for Systematic Reviews and Meta-Analyses (PRISMA)-compliant systematic review methodology to provide reproducible evidence summaries [102].

## Conclusions

AF arises from a diverse and mechanistically interconnected array of molecular perturbations spanning ion channel dysfunction, structural protein abnormalities, transcriptional dysregulation, and calcium handling defects. The four-module framework presented here, ion channelopathies (*SCN5A, KCNQ1, KCNH2, KCNA5*), structural myopathies (*TTN, MYL4, GJA5, COL1A1*), transcriptional regulators (*PITX2, GATA4, TBX5, NKX2-5, GATA6, ZFHX3*), and calcium handling proteins (RYR2, CASQ2, JPH2, ANKB, PLN, TRDN) provides an organizing lens for understanding how genetically heterogeneous mechanisms converge on a single arrhythmia phenotype. Ion channel mutations predominantly produce early-onset AF through direct disruption of action potential dynamics and are identifiable through currently available targeted gene panels in appropriate clinical settings. Structural and transcriptional mutations create the substrate for persistent arrhythmia through atrial remodeling, with TTNtv representing the most prevalent individually identifiable genetic cause. Calcium handling mutations generate triggered activity that initiates AF episodes across both channelopathy and structural disease contexts.

Clinically, the transition from phenotype-driven to genotype-informed management offers the promise of precision therapies tailored to individual molecular defects. Targeted genetic testing is currently clinically actionable in patients with early-onset or familial AF, enabling cascade screening, risk stratification, and, in selected cases, genotype-guided therapy selection. Polygenic risk scores, pharmacogenomics, and gene-specific therapeutic strategies represent near-term translational opportunities, though the persistent heritability gap and limitations of current functional models demand continued investment in basic research. Expansion of genetic studies to ethnically diverse populations and integration of multi-omic data with carefully validated machine learning approaches will be essential to fully realize the translational impact of AF genetics. All forward-looking statements in this review represent research priorities rather than established clinical practice.
